# The mediating role of coping behavior on the age-technostress relationship: A longitudinal multilevel mediation model

**DOI:** 10.1371/journal.pone.0213349

**Published:** 2019-03-05

**Authors:** Nathalie Hauk, Anja S. Göritz, Stefan Krumm

**Affiliations:** 1 Department of Psychological Assessment, Differential and Personality Psychology, Free University of Berlin, Berlin, Germany; 2 Department of Occupational and Consumer Psychology, University of Freiburg, Freiburg, Germany; Nathan S Kline Institute, UNITED STATES

## Abstract

This study seeks to explain the interplay between chronological age and technology-related strain through techno-stressors and coping strategy choices in organizational settings. Grounded in Lazarus´ stress theory, theories of cognitive aging, the life span theory of control and socioemotional selectivity theory, this study argues that even though older workers are more prone to techno-stressors, aging is connected to gaining coping skills, which in turn reduce technology-related strain over time. Understanding these processes enables modifying employees’ coping strategy choices and mitigating negative outcomes of technostress at the workplace. Longitudinal data from 1,216 employees over a time period of 8 months were used to perform multilevel mediation modeling. The findings reveal that age was negatively related to technology-related strain. The link between age and technology-related strain was explained through behavioral disengagement, which older workers used less than younger workers. Active coping and social coping did not act as mediators of this relationship across time points. These relationships were stable after controlling for dependency on technology.

## Introduction

The increasing number of older employees in the workforce [1] as well as the ongoing digitization of work [2] raise the question of how chronological age relates to stress arising from information and communication technology (ICT) usage in occupational settings. Despite empirical research endeavors on technology-related stress (hereinafter referred to as *technostress*), our understanding of the relationship between chronological age and technostress is limited. Research has shown that ICT dependency at work might induce stress, work exhaustion [3] and several negative consequences for the organization [4–6]. Despite perceiving technologies as useful, older adults have greater difficulty handling them [7]. Therefore, older workers might experience more situations in which they feel taxed by ICT-related demands. Yet as people age, they gain resources and coping abilities that help them deal with stress [8–10]. The future success of work organizations may depend on a deeper understanding of age-contingent differences in technostress management strategies. The identification of effective coping strategies buffering the consequences of technostress may be beneficial for developing organizational interventions to help employees cope more effectively with technology-related strain—especially as the proportion of older workers increases. Therefore, the current study aims to answer the research question: How do workers cope with technostress across the age span in a digitalized work environment?

In the following sections, we introduce the transactional theory of stress and its application to technostress, followed by an overview of different types of coping strategies. We then outline our theoretical rationale, first for age contingency in the experience of technology-related strain, second for age differences in applying coping strategies, and third for the mediating role of coping in the age-technostress relationship.

### Technostress and coping

Stress refers to an overall transactional process in which environmental demands exceed individuals’ capabilities and resources to meet them [11]. Following this notion, technostress is defined as a problem of adaptation due to an inability to cope with ICT-related demands [6, 12]. In the following paragraphs, the technostress formation process is illustrated on the basis of the transactional theory of stress [11].

According to the transactional theory of stress, stress is formed through three processes: primary appraisal, secondary appraisal, and coping. Primary appraisal is defined as the process of perceiving a threat to oneself: for example, a perceived misfit between technological demands and one´s own abilities to meet them [11]. Five factors have been identified as determinants of technostress (hereinafter referred to as *techno-stressors*) in occupational settings [6, 12]: Techno-overload refers to situations in which users feel forced by ICT to work faster and longer. Techno-invasion refers to situations in which work and private life merge due to constant availability and connectedness through ICT. Techno-complexity refers to situations in which users feel overwhelmed or incompetent in the face of ICT. Techno-insecurity refers to situations of perceived job insecurity due to the fear of being replaced by either ICT or by workers with greater ICT skills. Techno-uncertainty refers to situations of unpredictable, continuous ICT-related changes and upgrades.

Secondary appraisal is defined as the process of evaluating available coping resources to handle a perceived threat [11]. Hence, this process depends on an individual’s resources (e.g., coping competencies) as well as on environmental factors (e.g., circumstances). Generally, coping is defined as “adaptational acts that an individual performs in response to disruptive events that occur in his/her environment” [13]. Accordingly, coping includes problem-focused actions aimed at changing the stressful situation, for example, trying to gain control of the stressful situation step-by-step (*active coping)* or asking others for help (*seeking instrumental social support*). Other forms of coping include ignoring the stressful situation, denial, or disengagement: for example, breaking off any further interaction and withdrawing from the stressful situation (*behavioral disengagement*). Notably, individuals might engage in more than one strategy at a time [13, 14]. Therefore, coping strategies are not mutually exclusive, and individuals might not only differ in their choice of coping strategies, but also in the extent to which they engage in any single strategy. For example, according to recent research, almost all employees (88.3%) initially try to fix an ICT-related problem themselves or try to seek the source of the problem [15].

The third component of the stress formation process consists of executing the coping behaviors with the goal of reducing negative consequences [11]. Following common taxonomies of coping, we differentiate between functional and dysfunctional coping. Accordingly, behavioral disengagement leads the experienced strain level to decline, but it might rise again beyond the initial level if further encounters with the stressor cannot be avoided in the long run [16]. Therefore, behavioral disengagement is considered a dysfunctional coping strategy, whereas active coping and seeking instrumental social support are considered functional coping strategies [16]. A study on different coping strategies [17] examined three adaptive coping strategies in the technostress context (active coping, asking for technical support, and planning), as well as two maladaptive coping strategies (denial and behavioral disengagement). In this study, employees mostly reacted to technology-related strain with maladaptive coping strategies, which in turn increased work exhaustion, whereas adaptive coping reduced work exhaustion [17]. Hence, strain is not a direct result of experiencing a stressor but rather dependent on an individual’s resources and coping abilities. Indeed, personal resources, such as emotional and mental competencies, are negatively related to technology-related strain at work [18].

Furthermore, not only does coping behavior influence the experience of technology-related strain, the experience of technology-related strain initiates coping behavior [11]. Such feedback loops may cause individuals to continue to engage in coping behavior when they perceive the strain level as still too high. For example, Maier, Laumer, Weiner, and Weitzel [19] showed that techno-stressors led to technology-related exhaustion, and technology-related exhaustion caused behavioral responses such as avoidance.

Adverse consequences of technostress in individuals include decreased well-being [20], increased exhaustion [5, 21], and physiological stress reactions such as the release of stress hormones and increased blood pressure [22, 23]. In organizational settings, exposure to techno-stressors has been related to decreased job commitment [6, 12, 24, 25], lower usage continuance intention [19, 20], lower productivity [12, 26], lower job performance [4, 5], increased job stress [26], lower job satisfaction [6, 12, 25], increased risk of job burn-out, and decreased job engagement [27]. Considering the severity of these consequences for employees and organizations, it comes as a surprise that little research has focused on individual coping strategies to handle techno-stressors and consequently reduce technology-related strain. Furthermore, the few existing studies on coping with technostress used cross-sectional designs instead of evaluating long-term effects, despite the fact that coping is a dynamic process that unfolds over time. Therefore, the aim of this study is to investigate the mediating effects of coping over time.

In summary, technostress refers to perceived threatening situations involving ICTs that may result in technology-related strain. It is a contextual, dynamic process that varies depending on individuals’ resources and coping abilities. Feedback loops lead to reappraisal of the situation and potentially to modified coping behaviors. In the following section, we delineate the role of age within the technostress framework.

### Age and technostress

We rely on general aging theories to develop our hypotheses. We propose that two opposing mechanisms influence the relationship between age and technostress. On the one hand, aging is connected to physical degeneration processes, such as cognitive decline [28, 29], that make older adults more exposed to some but not all techno-stressors [30]. On the other hand, aging is connected to higher resilience, a broader repertoire of coping strategies and increased competence in handling emotions [31, 32]. We propose that such age-related gains lead to more efficient coping with techno-stressors and consequently decreased technology-related strain over time. In the following paragraphs, we unfold these arguments and propose our hypotheses.

#### The relationship between age and techno-stressors

Cognitive theories on aging center on cognitive decline (e.g., working memory, processing speed) across the life span [28, 29]. Further physical age-related degeneration processes include deterioration in hearing, vision and fine motor skills [33–35]. All of these physical skills are required to use ICTs. For instance, age effects on technostress might arise from declines in working memory, such as the capacity to process information needed to complete ICT-related tasks. Indeed, a meta-analysis on age and technology acceptance revealed that older adults report greater difficulties in handling technology compared to younger adults [7]. Two techno-stressors, namely techno-overload and techno-complexity, refer to situations specifically related to handling ICTs. In these situations, individuals’ cognitive abilities and physical condition are paramount to meeting technological demands. In contrast, the other three techno-stressors, namely techno-insecurity, techno-uncertainty, and techno-invasion, refer to situations in which cognitive abilities play a minor role in meeting ICT-related demands. Therefore, we do not expect all dimensions of technostress to be prone to age effects. On average, however, as people grow older and experience more difficulties handling technology, situations related to ICT usage should be appraised as threatening more often compared to younger individuals. We conclude that the relationship between age and level of techno-stressors overall should be positive due to physical age-related decline. We therefore hypothesize:

*H1*: *Age is positively correlated with techno-stressors*.

Hitherto empirical evidence on the relationship between age and techno-stressors has been mixed. In line with our theorizing are the results of one study in which age was positively correlated with techno-overload and techno-complexity, but not correlated with techno-invasion, techno-insecurity and techno-uncertainty [36]. Furthermore, most studies report that age is a significant positive predictor of how employees perceive the overall level of techno-stressors [20, 36–38]. However, one study reported that the experience of techno-stressors decreases as age increases [6]. However, those studies merely included age as a control variable and lack sufficient theoretical foundation. Moreover, most of the samples did not include age ranges representative of the working population and had only a small proportion of older workers [36, 38]. Therefore, hitherto findings on age effects should be treated with caution.

#### The relationship between age and coping

The life-span theory of control suggests that the use of primary control strategies increases with age until it declines after retirement, for example, at 65 years of age and older [9]. Primary control refers to changing the immediate environment and therefore relates to active problem-solving behavior [8, 11]. Thus, according to the life-span theory of control, chronological age should be connected to an increase in both active coping as well as seeking instrumental social support over the course of one’s working life. Furthermore, theories on life-span development suggest that aging is connected to gaining “situational, strategic, and procedural knowledge about emotional situations” [31], as well as accumulating knowledge about and experience with challenging situations [39, 40]. These gains in internal resources enable individuals to adopt problem-solving strategies that are more context-specific and more adaptive as they grow older [31, 40]. Hence, older adults make use of a broader repertoire of coping strategies when facing challenges [31, 39, 41, 42]. Moreover, according to socioemotional selectivity theory, emotionally gratifying experiences gain importance when one’s lifetime is perceived as increasingly limited, which is naturally the case during aging [41, 43]. This shift towards mood-enhancement goals should further increase older adults’ motivation to engage in successful coping. In summary, age effects in coping decisions are based on shifts in norms, motives, emotional goals and behavioral strategies over the course of adulthood [31].

Although coping options seem to be highly situational, the coping strategies of active coping, seeking instrumental social support, and behavioral disengagement are among the most prevalent coping strategies in the occupational technostress context [15]. Therefore, we will focus our hypotheses on these three strategies.

Active coping includes taking direct action as well as increasing one’s efforts to address a stressful situation [44]. Hence, active coping requires high motivation, strong problem-solving skills, and task-related knowledge–all of which are more pronounced with increasing age. Furthermore, empirical evidence suggests that older employees use more active coping when confronted with work-related stress compared to their younger colleagues [45]. Therefore, we hypothesize:

*H2a*: *Age is positively correlated with active coping*.

We propose that the ability to seek instrumental social support depends on a person’s capacity to interact with their social environment. More precisely, seeking instrumental social support involves asking for advice, seeking assistance, and gathering information from others [44]. Therefore, seeking instrumental social support depends on organizational interconnectedness as well as social and communication skills. Previous research has shown that aging is connected to increased social skills, for example, being more sensitive to emotional cues when making social inferences [46] and being more competent in managing social interactions [40]. Hence, building good interpersonal relationships with colleagues, supervisors, and experts in the organization might be easier for older workers compared to younger workers. Being able to rely on a strong social network provides the basis for engaging in social coping, for example, asking someone knowledgeable for help.

Furthermore, personality traits influence behavioral reactions to stressful events, including coping mechanisms [27, 47]. We argue that older workers rely on social coping more extensively than younger workers due to personality development. Research on personality development over the lifespan suggests that older individuals are more emotionally stable, agreeable, and conscientious [48]. Individuals with high levels of agreeableness are described as kind, considerate, likable and cooperative [49], have a communal orientation [50], are friendly, helpful, and empathetic [51]. Hence, individuals high in agreeableness might be more willing to be guided and more accepting of support by others. Therefore, we hypothesize:

*H2b*: *Age is positively correlated with seeking instrumental social support*.

Behavioral disengagement includes reducing one’s amount of effort and giving up the attempt to change a stressful situation [44]. Withdrawal from a conflict might be wise in interpersonal situations and is therefore considered a social skill that is more often used by older as compared to younger adults [40, 52]. However, while avoiding a stressor might be adaptive in interpersonal conflicts, avoidance is not an effective strategy when faced with techno-stressors. Insights drawn from personality psychology suggest that the average level of conscientiousness increases over the life-span [48]. Conscientiousness is reflected in individual behaviors such as being organized, tidy, reliable, and responsible [53]. The planning, disciplined component of the conscientiousness trait makes disengagement less likely [53]. Hence, increasing levels of conscientiousness might influence secondary appraisal processes such as the evaluation of appropriate coping strategies in the face of stressors. Indeed, empirical findings suggest that individuals with lower levels of responsibility were less likely to use instrumental coping and more likely to use avoidance [54]. We argue that older workers will less often disengage from techno-stressors in occupational settings because they will seek to act responsibly and feel obliged to do their duty. Moreover, behavioral disengagement is used more often when problem-oriented coping is not available [44]. However, since aging is connected to a broader variety of coping strategies, older workers might less often have to opt for alternative coping strategies. According to previous research, the use of functional coping strategies when faced with daily stressors increases over the course of adulthood, whereas the use of dysfunctional coping strategies decreases [55]. Consequently, we propose that older workers engage in less behavioral disengagement than younger workers when confronted with technostress at work due to higher conscientiousness. Corroborating findings suggest that older adults use more problem-focused coping when facing instrumental problems, and use more avoidant, denial-based coping when facing interpersonal problems [56]. Therefore, we hypothesize:

*H2c*: *Age is negatively correlated with behavioral disengagement*.

#### The relationship between age and technology-related strain

Most studies published so far have examined antecedents of technostress as well as work-related outcomes such as productivity. Studies on adverse psychological outcomes like technology-related strain are few and their results with regard to age are somewhat unclear [57–59]. Nevertheless, a systematic review of the relationship between ICT use and health outcomes concluded that older employees do not experience more stress or burnout than their younger colleagues when using ICT [60]. However, none of the studies included in the review formulated age-specific hypotheses.

Insights drawn from work and organizational psychology research suggest that older workers experience lower levels of emotional exhaustion than younger workers [61] and that perceived strain levels at work decline with age [62]. According to the life-span theory of control, aging is connected to an increase in secondary control strategies, such as cognitive coping strategies aimed at changing internal processes [8, 9]. Accordingly, aging is connected to increased self-regulation skills. Moreover, as people age they become more skilled in handling negative emotions [40] and have a focus on positive stimuli and emotional well-being [39, 41]. In addition, aging is connected to higher resilience to certain stressors [42, 63]. Higher self-regulation skills, more positive appraisals and higher resilience might explain why older adults report less technology-related strain at work. Therefore, we propose that even though reactivity to stressors exists among all age groups, the association is less strong among older adults [32]. Therefore, we hypothesize:

*H3*: *Age is negatively correlated with technology-related strain*.

Moreover, we suggest that coping strategies determine the level of technology-related strain that workers experience. Coping mechanisms are critical for outcomes such as psychological well-being [55]. Presumably, older workers’ lower level of work stress and technology-related strain is due to greater competence in handling stressors. For example, the use of more active coping was revealed to be an explanatory variable for the age-strain relationship in the work context [45]. This notion is substantiated by further research revealing that coping processes explain differences in work exhaustion as a consequence of techno-stressors [17]. Specifically, when dealing with techno-stressors, problem-focused coping reduces work exhaustion, whereas disengaging and ignoring the problem increase work exhaustion [17]. As proposed earlier, we suggest that older workers engage more in functional coping and less in dysfunctional coping on average. Furthermore, we propose that higher usage of functional coping leads to lower ICT-related strain over time, whereas higher usage of dysfunctional coping increases ICT-related strain over time. Finally, we suggest that coping style differences function as a buffer against age-related increases in techno-stressors and explain age effects on perceived technology-related strain. Therefore, we hypothesize:

*H3a*: *The correlation between age and technology-related strain is mediated through techno-stressors and functional coping (active and social coping)*.
*H3b*: *The correlation between age and technology-related strain is mediated through techno-stressors and dysfunctional coping (behavioral disengagement)*.

## Method

### Participants and procedure

We conducted a web-based study with the online panel WiSoPanel [64], which comprises members from German-speaking countries (Germany, Austria and Switzerland). Data were collected at three time points (i.e., T1, T2, T3), each separated by four months. Participants were contacted via email. The only selection criterion for participation was being regularly employed at all measurement points.

APA ethical standards were followed throughout the study. Approval from an ethics committee was not requested since the sample exclusively consisted of registered panelists who regularly take surveys like the one included in this study. All participants were informed about the purpose, method, content, and duration of the study. Furthermore, participants were informed about voluntariness of the participation, the organizations responsible for conducting the study, and contact persons. All study participants gave informed consent by clicking on a button.

A total of *N*_T1_ = 1,216 professionals completed the survey at the first measurement point, *N*_T2_ = 840 professionals responded again at the second measurement point, and *N*_T3_ = 631 professionals participated in all three measurement points. The mean age of the sample was 46.3 years (*SD* = 10.7), ranging from 17 to 75 years, and 55.2% of the participants were women. Participants were well-educated: Half of the participants had finished O-Levels or A-Levels (51.1%), and 40.4% had completed a university degree or higher, whereas 8.5% of the participants had no educational degree. Many participants worked in companies with more than 250 employees (*N* = 570, 46.9%), whereas 22.3% (*N* = 271) worked in companies with between 50 and 250 employees, and 30.8% (*N* = 375) worked in companies with up to 50 employees. More than 50% had worked in their company for more than 10 years, and 76.5% had received at least one ICT-related training so far. All participants received monetary compensation for completing the questionnaire at each measurement point.

### Measures

#### Techno-stressors

Technostress at the workplace was measured with 23 items published in Tarafdar, Tu, Ragu-Nathan, and Ragu-Nathan [12]. The items cover five techno-stressors: (1) techno-overload, defined as the feeling of being pressured to work faster and longer due to the implementation of ICT, for example, *“I am forced by this technology to work much faster”;* (2) techno-invasion, defined as permanent availability due to ICT usage, for example, *“I feel my personal life is being invaded by this technology”;* (3) techno-complexity, defined as a feeling of incompetence in the face of ICT, for example, *“I need a long time to understand and use new technologies”;* (4) techno-insecurity, defined as the feeling of being replaceable due to technological innovation, for example,”*I am threatened by coworkers with newer technology skills”;* and (5) techno-uncertainty, defined as the feeling that ICT is constantly changing, for example, *“There are constant changes in computer software in our organization*.*”* All items were measured on a Likert scale from 1 (*never*) to 5 (*always*). The scale showed high reliability across measurement points (α = .94 to .95; cf. S1 Table).

#### ICT-related coping

ICT-related coping behavior was measured using three scales (active coping, seeking instrumental social support, and behavioral disengagement) adapted from Carver, Scheier, and Weintraub [44]. To adjust the items to the ICT context, we gave participants the following instructions: “*Indicate to which extent your thoughts and actions correspond to the following statements about difficult or unpleasant situations handling ICT at work in the past*.” Ratings were given on a Likert scale from 1 (*never*) to 4 (*always*). Active coping was measured with 4 items, for example, “*I concentrate my efforts on doing something about it*.” Seeking instrumental social support was measured with 4 items, for example, “*I try to get advice from someone about what to do*.” Behavioral disengagement was measured with 4 items, for example, “*I admit to myself that I can´t deal with it and quit trying*.” Each scale showed high reliability across measurement points (α = .87 to .91; cf. S1 Table). For practical reasons, we will subsequently use the term social coping when referring to seeking instrumental social support.

#### ICT-related strain

ICT-related strain, including physical and emotional exhaustion, was measured as the degree to which an employee felt strained due to ICT usage in connection with work tasks. We used a 5-item scale adapted from Moore [65, 66] that included items such as “*I feel emotionally drained from ICT use at work*” or “*I feel fatigued from work assignments which involve the application of ICT*.” Items were rated on a Likert scale from 1 (*never*) to 7 (*always*). The scale showed high reliability across measurement points (α = .95 to .97; cf. S1 Table).

#### Control variables

Research has shown that the experience of ICT-related strain is not limited to any specific occupation [66]. Nevertheless, ICT usage varies across different occupations [21]. Since higher technology dependence among employees is associated with higher levels of technostress [37], we included ICT dependency for work-related tasks as a control variable. ICT dependency was measured with a 7-item scale [37], for example, “*It would be difficult to imagine my work without a computer*” or “*All of knowledge sharing and information transferring are carried out via internet or intranet in my organization*.” The statements were rated on a Likert scale ranging from 1 (*strongly disagree*) to 7 (*strongly agree*). The scale exhibited high internal consistency across measurement points (α = .89 to .92; cf. S1 Table).

### Analysis procedure

**Fig 1:**
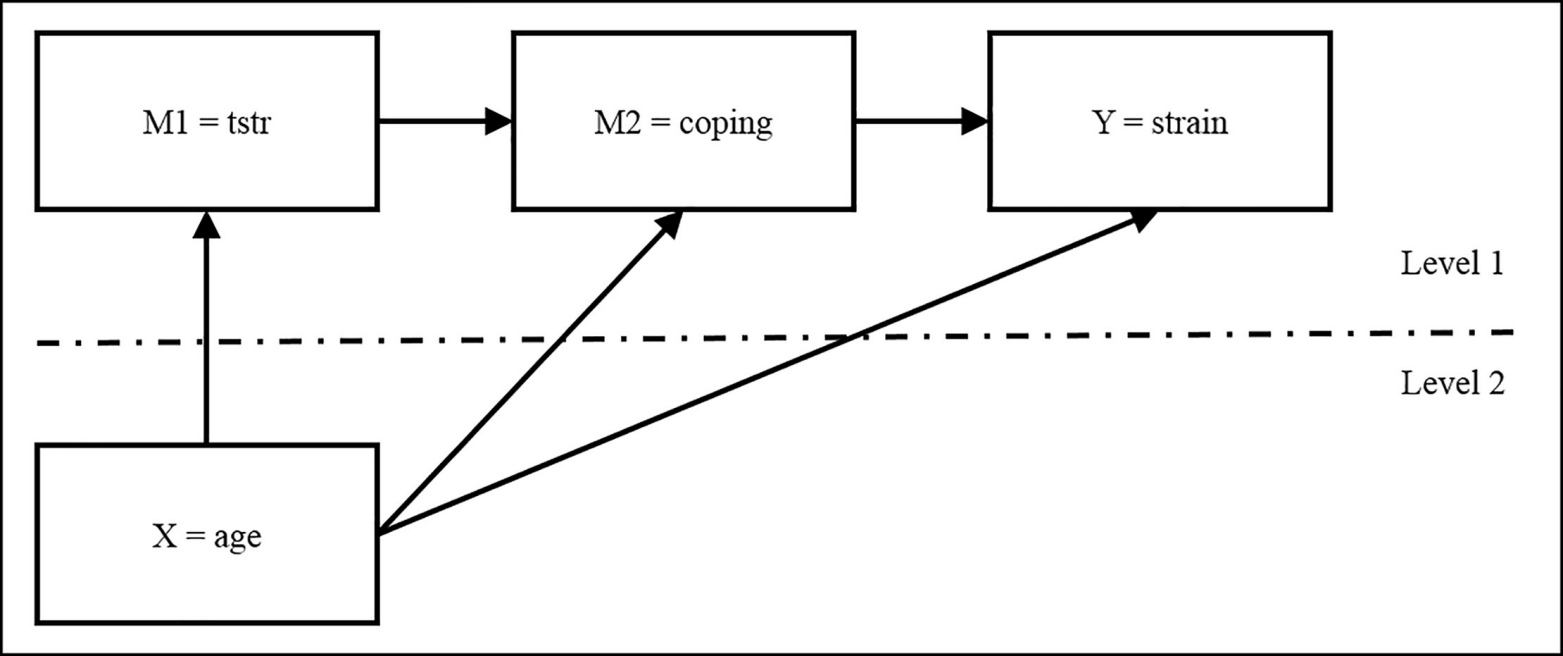
Research model. We additionally considered ICT dependency as a control variable in the model by including the direct effect of ICT dependency on all variables.

We used multilevel modeling (MLM) to account for data dependency due to repeated measurement in our longitudinal study. Moreover, MLM has the advantage of exploiting all valid data points in the analysis, therefore minimizing data loss due to attrition in longitudinal studies [67]. We fitted three separate 2-1-1-1 multilevel mediation models with two serial mediators each using Mplus version 7.1 [68]. Age was entered as the Level 2 predictor (X), techno-stressors (M1) and one coping strategy per model (M2) as Level 1 mediators and technology-related strain (Y) as the Level 1 outcome (cf. Fig 1). Effect parameters were based on maximum likelihood estimation. MLM within-level effects refer to individual changes over time, for example, how technology-related strain changes across measurement points as a function of coping. Between-level effects refer to inter-individual differences in these relationships, for example, how these processes differ across individuals of different ages.

#### Add-on analysis

We tested spiral effects–the specific temporal order of coping behavior and technology-related strain–using Model 6 of the SPSS PROCESS macro [69]. We conducted two separate serial mediation analyses with different starting points. First, we entered age as the predictor variable (X), technology-related strain at T1 as the first mediator (M1_T1_), coping strategies at T2 as the second mediator (M2_T2_) and technology-related strain at T3 as the outcome (Y_T3_). Second, we entered age as the predictor variable (X), coping strategies at T1 as the first mediator (M1_T1_), technology-related strain at T2 as the second mediator (M2_T2_) and coping strategies at T3 as the outcome (Y_T3_). We controlled for ICT dependency at T1 in both models.

## Results

Hypothesis 1 posited that chronological age and level of overall techno-stressors are positively related. Contrary to our hypothesis, age was negatively related to technostressors at T2 (*r*_T2_ = -.10), and not related to technostressors at T1 and T3 (*r*_T1_ = -.05, *r*_T3_ = -.06; cf. S1 Table). Furthermore, the multilevel mediation analysis yielded a significant negative total effect (β = -.07 [-.13; -.02], *p* < .05; cf. Table 1), though the effect of age on the level of overall techno-stressors was not significant (β = -.04 [-.09; .01], *p* = .224; cf. Table 1) when controlling for ICT dependency. Thus, Hypothesis 1 was not supported.

**Table 1:** MLM results on age and techno-stressors.

**Outcome: tstr**	Estimate	SE	*p*	LL	UL	*R*^2^	*p*
age	-.037	.031	.226	-.088	.013	.126	< .001
dep	.349	.029	.000	.300	.396		
**Total Effect**							
**age to tstr**	Estimate	SE	*p*	LL	UL		
total	-.073	.032	.023	-.125	-.020		

*Note*. Standardized model results, *N* = 1,216, SE = standard error, *p* = two-tailed *p*-value, LL/ UL = 95% lower-level and upper-level confidence interval, tsrt = techno-stressors, dep = ICT dependency

Hypothesis 2a stated that chronological age and the use of active coping are positively related. Contrary to our hypothesis, age was negatively related to active coping at T1 (*r*_T1_ = -.10) and not related to active coping at T2 and T3 (*r*_T2_ = -.02, *r*_T3_ = -.02; cf. S1 Table). Drawing on the multilevel mediation results, there was a small yet significant negative total effect of age on active coping (β = -.09 [-.14; -.04], *p* < .05; cf. Table 2). However, the direct effect of age on active coping was not significant when the level of techno-stressors and ICT dependency were controlled for (β = -.03 [-.01; .02], *p* = .286; cf. Table 2). Hence, active coping was not related to age. Thus, Hypothesis 2a was not supported.

**Table 2:** MLM results on age and active coping.

**Outcome: active**	Estimate	SE	*p*	LL	UL	*R*^2^	*p*
age	-.030	.029	.286	-.077	.017	.339	< .001
tstr	.020	.036	.568	-.038	.079		
dep	.571	.035	.000	.514	.628		
**Total Effect**							
**age to active**	Estimate	SE	*p*	LL	UL		
total	-.090	.032	.006	-.143	-.037		

*Note*. Standardized model results, *N* = 1,216, SE = standard error, *p* = two-tailed *p*-value, LL/ UL = 95% lower-level and upper-level confidence interval, tstr = techno-stressors, dep = ICT dependency

Hypothesis 2b stated that chronological age and the use of social coping are positively related. Contrary to our hypothesis, chronological age was negatively related to social coping at T1 and T2 (*r*_T1_ = -.13, *r*_T2_ = -.07), and unrelated at T3 (*r*_T3_ = -.05; cf. S1 Table). Drawing on the multilevel mediation results, there was a significant negative total effect of age on social coping (β = -.14 [-.20; -.09], *p* < .05; cf. Table 3). Furthermore, the direct effect of age on social coping was significant when the level of techno-stressors and ICT dependency were controlled for (β = -.09 [-.14; -.04], *p* < .01; cf. Table 3). Hence, social coping decreased as age increased. Hypothesis 2b was not supported.

**Table 3:** MLM results on age and social coping.

**Outcome: social**	Estimate	SE	*p*	LL	UL	*R*^2^	*p*
age	-.087	.030	.003	-.137	-.038	.284	< .001
tstr	.168	.035	.000	.110	.225		
dep	.433	.035	.000	.376	.490		
**Total Effect**							
**age to social**	Estimate	SE	*p*	LL	UL		
total	-.144	.032	.000	-.197	-.090		

*Note*. Standardized model results, *N* = 1,216, SE = standard error, *p* = two-tailed *p*-value, LL/ UL = 95% lower-level and upper-level confidence interval, tstr = techno-stressors, dep = ICT dependency

Hypothesis 2c posited that chronological age and the use of dysfunctional coping strategies, such as behavioral disengagement, are negatively related. In line with this hypothesis, we found a significant negative correlation between age and behavioral disengagement at all measurement points (*r*_T1_ = -.10, *r*_T2_ = -.14, *r*_T3_ = -.14; cf. S1 Table). This was further supported by the multilevel mediation analysis, which yielded a significant total effect (β = -.15 [-.21; -.10], *p* < .001; cf. Table 4), as well as a significant direct effect (β = -.11 [-.16; -.07], *p* < .001; cf. Table 4) after controlling for the level of techno-stressors and ICT dependency. Hence, behavioral disengagement decreased as age increased. Thus, Hypothesis 2c was supported.

**Table 4:** MLM results on age and behavioral disengagement.

**Outcome: diseng**	Estimate	SE	*p*	LL	UL	*R*^2^	*p*
age	-.114	.026	.000	-.156	-.072	.569	< .001
tstr	.782	.025	.000	.741	.823		
dep	-.168	.030	.000	-.217	-.119		
**Total Effect**							
**age to diseng**	Estimate	SE	*p*	LL	UL		
total	-.153	.034	.000	-.210	-.097		

*Note*. Standardized model results, *N* = 1,216, SE = standard error, *p* = two-tailed *p*-value, LL/ UL = 95% lower-level and upper-level confidence interval, tstr = techno-stressors, dep = ICT dependency

Hypothesis 3 stated that chronological age and technology-related strain are negatively related. In line with this hypothesis, there was a significant negative correlation between age and technology-related strain at all three measurement points (*r*_T1_ = -.08, *r*_T2_ = -.14, *r*_T3_ = -.13; cf. S1 Table). Furthermore, the results of the multilevel mediation analysis indicated a significant negative total effect of age on technology-related strain (β = -.12 [-.18; -.07], *p* < .05). Hence, technology-related strain decreased as age increased. Hypothesis 3 was supported.

Hypothesis 3a posited that functional coping strategies, such as active problem solving and social coping, mediate the negative relationship between chronological age and technology-related strain. In contrast to this hypothesis, there was no significant indirect effect of age on technology-related strain via active coping (β = -.00 [-.00; .00], *p* = .753; cf. Table 5), via techno-stressors (β = -.03 [-.09; .01], *p* = .224; cf. Table 5), or via both mediators (β = .00 [.00; .00], *p* = .748; cf. Table 5). Hence, we found no evidence that active coping is a resolving link in the relationship between age and technology-related strain, as already indicated by the non-significant bivariate correlations at T2 and T3 (cf. S1 Table).

**Table 5:** Age effects on technology-related strain via techno-stressors and active coping.

**Outcome: strain**	Estimate	SE	*p*	LL	UL	*R*^2^	*p*
age	-.068	.024	.004	-.106	-.029	.618	< .001
tstr	.790	.021	.000	.756	.824		
active	.012	.036	.742	-.048	.072		
dep	-.043	.033	.200	-.097	.012		
**Total, Direct and Indirect Effects**							
**age to strain**	Estimate	SE	*p*	LL	UL		
total	-.122	.033	.000	-.176	-.068		
total indirect	-.054	.025	.029	-.095	-.013		
via tstr	-.029	.024	.224	-.069	.010		
via active	.000	.001	.753	-.002	.002		
via tstr and active	.000	.000	.748	.000	.000		
direct effect	-.068	.024	.004	-.106	-.029		

*Note*. Standardized model results, *N* = 1,216, SE = standard error, *p* = two-tailed *p*-value, LL/ UL = 95% lower-level and upper-level confidence interval, active = active coping, tstr = techno-stressors, dep = ICT dependency

Furthermore, there was no significant indirect effect of age on technology-related strain via social coping (β = -.00 [-.01; .00], *p* = .394; cf. Table 6), via techno-stressors (β = -.03 [-.09; .01], *p* = .222; cf. Table 6), or via both mediators (β = .00 [.00; .00], *p* = .447; cf. Table 6). Hence, there was no evidence that social coping explains the relationship between age and technology-related strain. Thus, Hypothesis 3a was not supported.

**Table 6:** Age effects on technology-related strain via techno-stressors and social coping.

**outcome: strain**	Estimate	SE	*p*	LL	UL	*R*^2^	*p*
age	-.065	.024	.006	-.104	-.026	.618	< .001
tstr	.785	.021	.000	.750	.820		
social	.030	.033	.372	-.025	.084		
dep	-.049	.029	.097	-.049	.000		
**Total, Direct and Indirect Effects**							
**age to strain**	Estimate	SE	*p*	LL	UL		
total	-.122	.033	.000	-.176	-.068		
total indirect	-.057	.025	.022	-.097	.016		
via tstr	-.029	.024	.222	-.069	.010		
via social	-.003	.003	.394	-.008	.002		
via tstr and social	.000	.000	.447	-.001	.000		
direct	-.065	.024	.006	-.104	-.026		

*Note*. Standardized model results, *N* = 1,216, SE = standard error, *p* = two-tailed *p*-value, LL/ UL = 95% lower-level and upper-level confidence interval, social = social coping, tstr = techno-stressors, dep = ICT dependency

Hypothesis 3b stated that chronological age leads to less dysfunctional coping, which in turn leads to less technology-related strain. In line with this hypothesis, there was a significant indirect effect of age on technology-related strain via behavioral disengagement (β = -.04 [-.06, -.02], *p* < .001; cf. Table 7), but not via techno-stressors (β = -.02 [-.05, -.01], *p* = .226; cf. Table 7), or via both mediators (β = -.01 [-.02, .00], *p* = .245; cf. Table 7). Interestingly, the direct path from age to technology-related strain was no longer significant (β = -.03 [-.07; .01], *p* = .175; cf. Table 7) when taking indirect effects into account. This indicates full mediation of the relationship between age and technology-related strain through behavioral disengagement. Hypothesis 3b was supported.

**Table 7:** Age effects on technology-related strain via techno-stressors and behavioral disengagement.

**outcome: strain**	Estimate	SE	*p*	LL	UL	*R*^2^	*p*
age	-.030	.022	.175	-.067	.007	.665	.000
tstr	.531	.050	.000	.449	.613		
diseng	.331	.055	.000	.241	.421		
dep	.021	.024	.380	-.018	.059		
**Total, Direct and Indirect Effects**							
**age to strain**	Estimate	SE	*p*	LL	UL		
total	-.122	.033	.000	-.176	-.068		
total indirect	-.091	.027	.001	-.136	-.046		
via tstr	-.020	.016	.226	-.046	.007		
via diseng	-.038	.011	.000	-.055	-.020		
via tstr and diseng	-.010	.008	.245	-.023	.004		
direct	-.030	.022	.175	-.067	.007		

*Note*. Standardized model results, *N* = 1,216, SE = standard error, *p* = two-tailed *p*-value, LL/ UL = 95% lower-level and upper-level confidence interval, diseng = behavioral disengagement, tstr = techno-stressors, dep = ICT dependency

**Fig 2:**
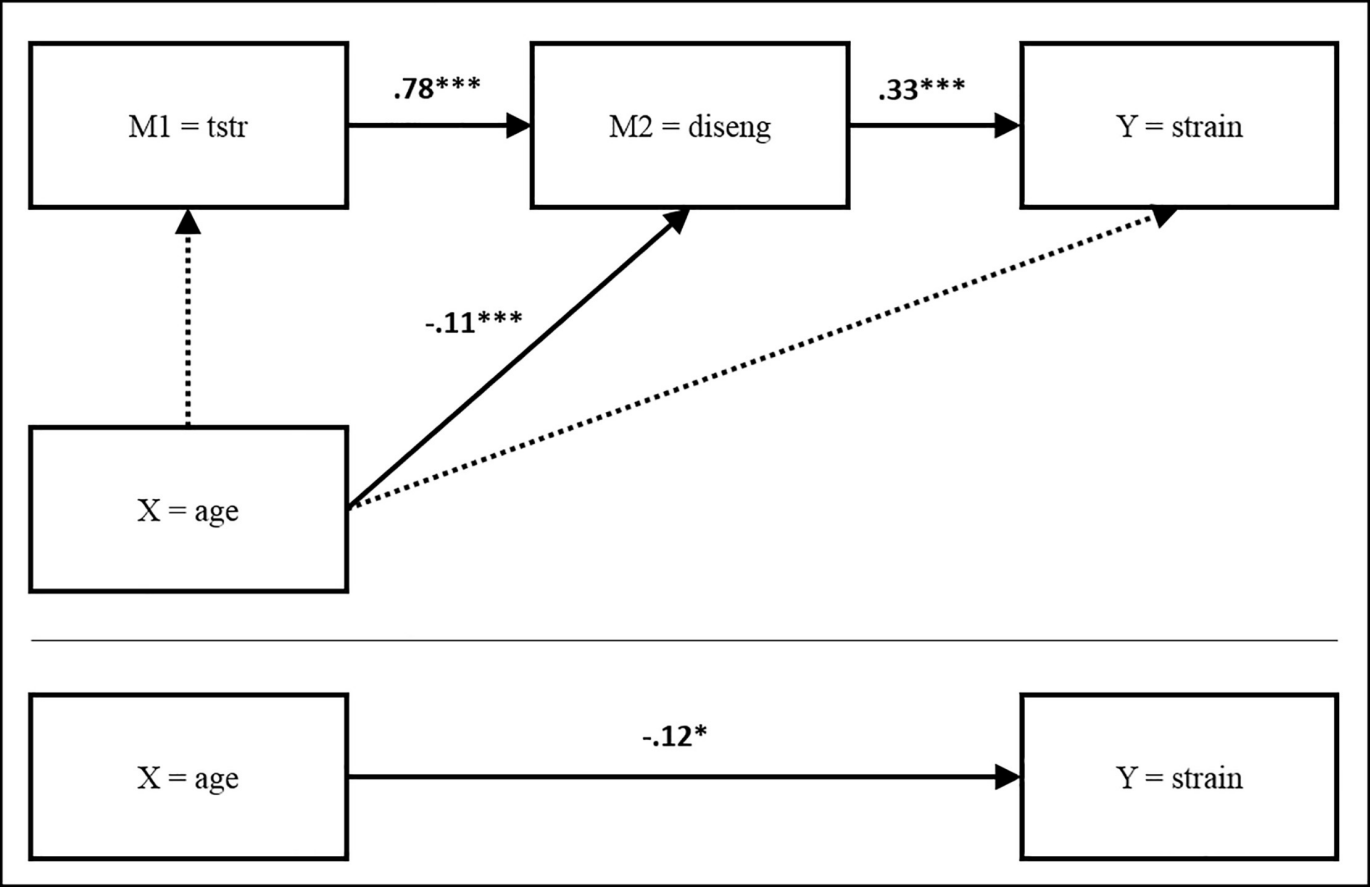
Model with behavioral disengagement. Standardized path coefficients are given in S4 Table; the dotted lines indicate non-significant effects; ICT dependency was considered as a control variable in the model by including the direct effect of ICT dependency on all variables.

In summary, age effects were driven by negative direct effects of age on behavioral disengagement (β = -.11 [-.17; -.07], *p* < .001; cf. Table 4). Hence, the negative association between age and behavioral disengagement buffered what was otherwise an amplification of the stress process from techno-stressors to increased behavioral disengagement (β = .78 [.74; .82], *p* < .001; cf. Table 4), and from behavioral disengagement to increasing technology-related strain (β = .33 [.24; .42], *p* < .001; cf. Table 7, Fig 2).

Furthermore, within-level effects reflect the development of the technostress process over time: The positive relationship between techno-stressors and technology-related strain (β = .30 [.24; .36], *p* < .001) was partly mediated by active coping (β = .01 [.00; .01], *p* < .05; cf. Table 8), social coping (β = .01 [.00; .02], *p* < .05; cf. Table 8), and behavioral disengagement (β = .03 [.02; .05], *p* < .001; cf. Table 8). Hence, coping strategies partially explain the development of the stress process from techno-stressors to technology-related strain over time. Specifically, engagement in any coping activity enhanced the technology-related strain level across measurement points.

**Table 8:** Coping effects on technology-related strain over time.

trs to strain		Estimate	SE	*p*	LL	UL
total		.299	.034	.000	.244	.355
A	direct	.292	.034	.000	.237	.347
	via active	.007	.004	.035	.002	.013
B	direct	.290	.034	.000	.235	.346
	via social	.009	.004	.025	.003	.016
C	direct	.266	.032	.000	.213	.319
	via diseng	.033	.010	.001	.017	.049

*Note*. Standardized model results, A = mediation model with active coping as mediator, B = mediation model with social coping as mediator, C = mediation model with behavioral disengagement as mediator, SE = standard error, *p* = two-tailed *p*-value, LL/ UL = 95% lower-level and upper-level confidence interval, active = active coping, social = social coping, diseng = behavioral disengagement, tstr = techno-stressors, strain = technology-related strain

### Add-on analysis

To test spiral effects of behavioral disengagement and technology-related strain, we tested two separate serial mediation models with different starting points. The first model assumed that technology strain at T1 and behavioral disengagement at T2 mediate the relationship between age and technology strain at T3. The indirect effect of age on technology strain at T3 via the two mediators (age → strain T1 → diseng T2 → strain T3) was not significant (β = -.001, 95% CI [-.002; .000]; cf. S5 Table).

The second model assumed that behavioral disengagement at T1 and technology strain at T2 mediate the relationship between age and behavioral disengagement at T3. Indeed, the indirect effect of age on behavioral disengagement at T3 via the two mediators (age → diseng T1 → strain T2 → diseng T3) was significant (β = -.001, 95% CI [-.002; -.000]; cf. S5 Table).

In summary, the age effect on the development of the stress process seems to be a spiral effect within which not only does behavioral disengagement lead to increased technology-related strain, but technology-related strain leads to increased behavioral disengagement as well.

## Discussion

The goal of this longitudinal study was to evaluate how workers across the age-span cope with technostress in a digitalized work environment. Specifically, we conducted a series of multi-level mediation analyses to evaluate longer-term effects of coping on technology-related strain as well as age effects on the technostress process.

In line with our theorizing, we found that older workers reported a lower level of ICT-related strain. In contrast to our hypothesis, however, older workers engaged in less problem-focused coping, as indicated by negative total effects of age on active coping and social coping. As hypothesized, older workers exhibited less behavioral disengagement. Furthermore, any coping behaviors in response to technostress increased technology-related strain over time. The negative relationship between workers’ age and their technology-related strain was explained by less behavioral disengagement with increasing age, and not–contrary to our hypothesis–by more active or social coping. Additionally, spiral effects of age on technology-related strain and coping indicated that behavioral disengagement led to increased technology-related strain, and subsequently, technology-related strain led to increased behavioral disengagement.

Finally, and again in contrast to our assumptions, there was no significant relationship between age and the level of techno-stressors after controlling for job-related ICT dependency. Therefore, in occupational settings higher age is not per se connected to an increased prevalence of situations in which ICT-related demands exceed workers’ abilities to meet them.

The present study extends earlier work in several ways. First, most studies so far have merely added age as a control variable to their models and lack theoretical underpinnings for the role of age [6, 38, 70]. That is why we built on general aging theories to provide a previously missing theoretical foundation for the role of age and age-contingent coping in the technostress process. Second, little research has focused on individual coping strategies to handle techno-stressors and consequently reduce technology-related strain. Third, we extend earlier work by examining the dynamic processes of technostress and coping over a broad time window instead of relying on cross-sectional data. Fourth, most previous studies on technostress in occupational settings have focused on specific occupational groups [65]. However, to understand the general mechanisms of ICT-related strain at work, our sample consists of workers from various professions. Fifth, unlike previous studies, our sample covers the complete age span of workers, including workers of higher ages.

### Theoretical implications

The results of our study have several implications. First, the relationship between age and techno-stressors may be more complex than previously thought. While we built our theorizing on age-related cognitive and physical decline and argued that age would be positively related to techno-stressors [20, 36–38], older and younger individuals in fact experienced a similar number of stressful situations connected to ICT use in their working lives. Since we controlled for ICT dependency, similar levels of subjectively perceived technostress cannot be due to older workers’ reduced exposure to technology. Notably, technostress not only occurs when actually handling ICTs, but also due to ICT-related feelings of uncertainty, insecurity and invasion of one’s private life. These dimensions might be less strongly related to age than the actual handling of ICTs. For example, the techno-stressor insecurity–the fear of losing one’s job due to ICTs–might even be appraised as less threatening as workers approach retirement age [41]. Furthermore, greater work-related knowledge and experience might enable older workers to appraise situations related to uncertainty and the invasion of private life as less threatening. Rauschenbach and colleagues [62] showed that perceptions of work-related demands depend on the particularities of the situation. For example, if the demanding situation requires crystallized knowledge and experience, older workers may have an advantage compared to younger workers. Therefore, negative and positive age effects on the individual techno-stressors might balance each other out. Consequently, the overall level of techno-stressors might be unrelated to age.

Second, our results do not fully align with common perspectives on the effectiveness of coping strategies. On the one hand, in line with our findings, ongoing behavioral disengagement is believed to increase the level of technology-related strain over time [16]. On the other hand, contrary to our hypotheses, the results indicate that ongoing active coping and social coping subsequently increase technology-related strain as well. Despite being considered *functional* coping strategies, it seems that engaging in problem-focused coping over a longer period of time (in our case eight months) increases technology-related strain. One implication may be that a distinction needs to be drawn between coping with momentary stressors that can actively be addressed and reduced, and longer-lasting stressors that have an ongoing impact on one’s life. Indeed, the need to maintain coping activities over a longer stretch of time might draw on individual resources, require mental effort and energy, and consequently increase rather than reduce experienced strain.

Third, according to our findings, there is no direct relationship between age and ICT-related active coping and a negative direct relationship between age and ICT-related social coping. One implication is that coping-related age trajectories [55] may not generalize across different domains of coping. Perhaps the assumption of general age-related gains in coping competencies [31, 39, 41, 42] must be viewed in a more differentiated manner with respect to the technostress domain. For example, age-related gains in internal resources, such as knowledge about and experience with emotional situations [31], might not be specific enough when it comes to handling instrumental problems. Behaviors enabling active problem-solving, such as trying to control the stressful situation and doing what needs to be done, require specific task-related knowledge. ICT-related knowledge gains, however, might be unrelated to age, meaning that active coping with techno-stressors might not be associated with age either.

Another possible explanation might be found in the energy conservation model by Aldwin and Levenson [42], which seeks to explain age differences in coping strategies. According to this model, younger adults have more energy to spend than older adults and therefore engage more in problem-focused coping. Due to a lack of experience, however, they are not necessarily more efficacious in doing so than older adults. Older adults, by contrast, have greater knowledge about which strategies are efficacious. To use their energy more economically, older adults avoid ineffective coping activities. Thus, age is related to a decreased level of coping. However, despite engaging in less coping, older adults’ ability to effectively cope does not decline. In fact, research suggests that older adults engage less in coping strategies than younger adults; however, older adults still remain more effective in coping than younger adults [42, 54]. Following this notion, workers’ age might be a negative direct predictor of the extent of coping behaviors such as seeking instrumental social support.

Fourth, the results of our study imply that age-related gains in dealing with technostress are driven by decreased dysfunctional coping rather than increased functional coping. One might argue that the reason for lower levels of behavioral disengagement is that the average level of conscientiousness increases over the life-span [48]. Therefore, older workers less often disengage from techno-stressors due to feelings of responsibility and obligation in occupational settings. Hence, increasing levels of conscientiousness influence secondary appraisal in the evaluation of appropriate coping behaviors. Even though older workers engage in less functional coping compared to younger workers, they profit from decreased engagement in dysfunctional coping, as seen through their lower technology-related strain. This notion is supported by previous studies suggesting that dysfunctional strategies have a stronger influence on adverse outcomes than functional coping [17].

Fifth, older workers are often confronted with negative stereotypes [71, 72]. For example, stereotypes related to technology use include the belief that information technology jobs are not appropriate for older workers and that older workers are less willing to keep up with technology [73]. However, our results contradict these stereotypes, by showing that i) age is unrelated to the amount of techno-stressors encountered during working life, and ii) the relationship between age and ICT-related strain is actually negative. Thus, older workers experience less ICT-related strain than younger workers. Similar conclusions were drawn from the results of a meta-analysis on age and technology acceptance [7]. The authors suggest that the negative stereotype of technophobic older adults is unwarranted.

### Practical implications

Multiple studies have demonstrated the severe consequences of technostress for employees and organizations, including lower productivity [12, 26], lower job performance [4, 5], increased work exhaustion [5, 21], and increased risk of burnout [27]. Furthermore, technostress impacts the human body by triggering physical stress reactions such as the release of stress hormones and increased blood pressure [22, 23]. Understanding the association between workers’ age and daily work stressors such as technostress provides an avenue for developing preventive measures to lower the risk of age-related diseases, such as cardiovascular diseases. In particular, communicating effective and ineffective ways of coping as part of organizational support measures could help to improve employees’ health and maintain their well-being. Furthermore, our research shows that workers with higher IT dependency are at greater risk of encountering techno-stressors. Therefore, preventive measures might be particularly essential in occupations with heavy IT use.

### Future research

Further research might wish to shed some light on the qualitative aspects of social coping. Our study suggests that i) extensive social coping increases technology-related strain over time, and ii) older workers use less instrumental social support in the face of ICT-related stress. However, when it comes to examining age-related changes in stress and coping, qualitative aspects might be more revealing than the quantitative extent of coping. As previous research suggests, older adults remain as effective in coping as younger adults despite engaging in less coping, regardless of the pattern of coping strategies used [42, 54]. Therefore, future studies should examine not only the extent of social coping, but also its quality. One crucial component of seeking instrumental social support might be having access to the “right” person in the organization, such as an expert on ICT-related problems. It is possible that older workers profit from greater organizational knowledge and larger work-related networks, and are therefore more efficient in social coping. Furthermore, it could be revealing to examine social patterns of coping, that is, who is turning to whom when seeking social support. For example, are older workers more likely to turn to peers, experts, or supervisors compared to younger workers?

Furthermore, coping strategies other than the ones examined in this study might be considered in future research. Studies suggest that emotion-focused coping increases when individuals have limited control over the stressful situation [14]. This might especially hold true in occupational settings, in which the use of certain ICTs is mandatory [13]. Furthermore, the life-span theory of control suggests that the use of secondary control strategies such as emotion regulation increases throughout the life-span [8, 10]. Consequently, the negative relationship between age and technology-related strain might be due to internal emotion regulation processes. Two coping strategies, namely seeking emotional social support and cognitive reappraisal, could potentially contribute to handling ICT-related stressors in the work context [74]. Both strategies focus on changing the self in order to adjust to a stressful situation rather than changing the situation itself [53]. The former does so through emotional support and reassurance–instead of instrumental help [53]. Cognitive restructuring strives to change the self by changing one’s perception of the stressful situation, for example, by focusing on positive aspects of the situation [10]. Additional socio-emotional coping behaviors discussed in the technostress context are venting and co‐rumination [74].

Moreover, future research might consider age effects on techno-stressors in greater detail. As the theory section of this paper suggested, age effects might be pertinent for some dimensions of technostress but not for others. We argued that age effects are driven by physical degeneration processes [28, 29]. However, our results revealed no age effects on the level of overall techno-stressors. Examining the influence of age on the individual techno-stressors might extend our understanding of the role of age in the occupational technostress process.

Finally, future research might wish to use alternative strain measures. For example, experience-based measures assess individuals’ experiences in situ. Therefore, experience-based measures are less affected by generalized self-perceptions, social norms and stereotypical attitudes [75]. Furthermore, objective measures such as biological stress indicators might complement evidence based on self-reported data. Self-reports depend on appraisal processes and might therefore be biased by older workers’ decreased willingness to report psychological symptoms and negative emotions [31, 39]. So far, there are few studies applying objective stress measures, such as cardiovascular activity or levels of stress hormones, in the technostress context. An overview of the biology of technostress can be found in Riedl [76].

### Limitations

This study is not without limitations. First, the longitudinal design of our study can be viewed as both an advantage and a limitation. Relatively long time lags, as was the case in this study (4 months), allow for a long-term investigation of workers’ technostress, but are unsuited to capturing workers’ daily experiences. Therefore, our results are limited to mediated reactions to techno-stressors and coping reactions over time.

Second, we conducted a study about ICT-related strain that relied on the very medium of ICT. We elected to use an online panel in order to incorporate a broad range of occupations and age groups. However, workers with acute levels of technology-related strain are probably less likely to participate in surveys conducted through ICTs. Therefore, the results of our study could be biased by self-selection processes.

Third, further control variables could be included in the analyses. We included ICT-related dependency as a control variable. However, other variables that we did not assess in this study might be influential in the development of the technostress process as well. For example, a series of situational factors have been identified as stress-inhibiting variables, including the provision of IT support, organizational IT trainings, and employee involvement in the facilitation of new ICTs [4, 6]. These technostress inhibitors are thought to reduce the level of techno-stressors and, in turn, technology-related strain. Although situational factors within the organizational environment are expected to be unrelated to age, some studies have found a negative relationship between age and technostress inhibitors [20]. Thus, technostress inhibitors might be also considered as a control variable.

#### Conclusion

By conducting a longitudinal study on coping strategies within the age-technostress process, we revealed that age is negatively related to ICT-related sources of technostress as well as to ICT-related strain. The negative relationship between age and ICT-related strain was mediated through behavioral disengagement, which was more prevalent among younger than among older workers. Moreover, age was negatively related to the coping strategies of active coping and seeking instrumental social support. In sum, the findings speak against the assumption that older workers are more prone to technology-related stress at work.

## Supporting information

S1 Table Correlation matrix and descriptive statistics. (PDF)

S2 Table Results from MLM with techno-stressors and active coping as mediators. (PDF)

S3 Table Results from MLM with techno-stressors and social coping as mediators. (PDF)

S4 Table Results from MLM with techno-stressors and behavioral disengagement as mediators. (PDF)

S5 Table Spiral Effects: Results from two serial mediation models. (PDF)
