# Supplementary material for: The mediating role of coping behavior on the age-technostress relationship: A longitudinal multilevel mediation model
Source: PLoS One. 2019 Mar 5;14(3):e0213349. doi: 10.1371/journal.pone.0213349 (PMC6400396; doi:10.1371/journal.pone.0213349)
Supplement: S1 Table — (PDF) [file pone.0213349.s001.pdf]

S1 Table. Correlation Matrix and Descriptive Statistics.

					active			social			diseng			strain			tstr			dep	
	M	*SD*	α	age	t1	t2	t3	t1	t2	t3	t1	t2	t3	t1	t2	t3	t1	t2	t3	t1	t2
age	46.29	10.65																			
t1active	2.72	.78	.87	-.101**																	
t2active	2.68	.79	.89	-.018	.548**																
t3active	2.67	.80	.88	-.024	.600**	.541**															
t1social	2.57	.81	.90	-.131**	.755**	.488**	.478**														
t2social	2.53	.80	.89	-.072*	.481**	.767**	.456**	.597**													
t3social	2.48	.82	.90	-.045	.504**	.461**	.747**	.591**	.551**												
t1diseng	1.61	.62	.86	-.101**	.074*	.030	-.014	.215**	.161**	.100*											
t2diseng	1.60	.61	.85	-.143**	.049	.107**	-.042	.130**	.250**	.038	.595**										
t3diseng	1.61	.66	.87	-.138**	.022	.025	.113**	.115**	.148**	.220**	.556**	.550**									
t1strain	1.89	1.27	.95	-.083**	.143**	.131**	.126**	.213**	.213**	.179**	.519**	.490**	.425**								
t2strain	1.97	1.33	.96	-.143**	.120**	.147**	.126**	.184**	.236**	.197**	.457**	.558**	.469**	.723**							
t3strain	2.00	1.38	.97	-.125**	.130**	.098*	.177**	.206**	.207**	.237**	.458**	.470**	.535**	.721**	.729**						
t1tstr	13.69	4.71	.94	-.052	.173**	.169**	.160**	.254**	.273**	.208**	.555**	.481**	.455**	.650**	.591**	.597**					
t2tstr	13.96	4.86	.94	-.103**	.179**	.211**	.116**	.249**	.283**	.204**	.485**	.535**	.461**	.588**	.676**	.588**	.767**				
t3tstr	14.01	4.99	.95	-.063	.125**	.153**	.197**	.199**	.229**	.271**	.471**	.489**	.545**	.554**	.572**	.634**	.745**	.759**			
t1dep	3.68	1.05	.90	-.094**	.438**	.382**	.398**	.372**	.323**	.340**	.108**	.077*	.096*	.219**	.195**	.225**	.339**	.315**	.308**		
t2dep	3.69	1.10	.92	-.090**	.402**	.436**	.357**	.345**	.365**	.314**	.076*	.060	.063	.175**	.185**	.215**	.264**	.335**	.274**	.852**	
t3dep	3.65	1.10	.92	-.081*	.416**	.393**	.412**	.376**	.343**	.382**	.069	.064	.068	.173**	.190**	.218**	.252**	.279**	.339**	.825**	.841**

Note. Sample size = N_{t1} = 1,215, N_{t2} = 840, N_{t3} = 631, active = active coping, social = social instrumental support, diseng = behavioral disengagement, strain = technology-related strain, tstr = techno-stressors, dep = IT dependency, t1-t3 = measurement point 1-3, **Correlation is significant at the .01 level (2-tailed), *Correlation is significant at the .05 level (2-tailed).
