# Supplementary material for: The mediating role of coping behavior on the age-technostress relationship: A longitudinal multilevel mediation model
Source: PLoS One. 2019 Mar 5;14(3):e0213349. doi: 10.1371/journal.pone.0213349 (PMC6400396; doi:10.1371/journal.pone.0213349)
Supplement: S2 Table — (PDF) [file pone.0213349.s002.pdf]

S2 Table. Results from MLM with techno-stressors and active coping as mediators.

**Within Level**							
**Outcome: active coping**							
	Estimate	SE	*p*	LL	UL	R^2	*p*
tstr	.098	.031	.001	.048	.149	.024	.010
dep	.095	.030	.002	.045	.145		
**Outcome: technology-related strain**							
	Estimate	SE	*p*	LL	UL	R^2	*p*
tstr	.292	.034	.000	.237	.347	.092	.000
active	.076	.026	.004	.033	.119		
dep	-.026	.035	.452	-.083	.031		
**Between Level**							
**Outcome: techno-stressors**							
	Estimate	SE	*p*	LL	UL	R^2	*p*
age	-.037	.031	.224	-.088	.013	.125	.000
dep	.348	.029	.000	.300	.396		
**Outcome: active coping**							
	Estimate	SE	*p*	LL	UL	R^2	*p*
age	-.030	.029	.286	-.077	.017	.339	.000
tstr	.020	.036	.568	-.038	.079		
dep	.571	.035	.000	.514	.628		
**Outcome: technology-related strain**							
	Estimate	SE	*p*	LL	UL	R^2	*p*
age	-.068	.024	.004	-.106	-.029	.618	.000
tstr	.790	.021	.000	.756	.824		
active	.012	.036	.742	-.048	.072		
dep	-.043	.033	.200	-.097	.012		
**Total, Direct and Indirect Effects**							
**age to strain**							
	Estimate	SE	*p*	LL	UL		
total	-.122	.033	.000	-.176	-.068		
total indirect	-.054	.025	.029	-.095	-.013		
via tstr	-.029	.024	.224	-.069	.010		
via active	.000	.001	.753	-.002	.002		
via tstr and active	.000	.000	.748	.000	.000		
direct effect	-.068	.024	.004	-.106	-.029		

Note. Model was fit using Mplus 7.1. *N* = 1,216, SE = standard error, *p* = two-tailed *p*-value, LL/ UL = 95% lower-level and upper-level confidence interval, active = active coping, tstr = techno-stressors
