# Supplementary material for: The mediating role of coping behavior on the age-technostress relationship: A longitudinal multilevel mediation model
Source: PLoS One. 2019 Mar 5;14(3):e0213349. doi: 10.1371/journal.pone.0213349 (PMC6400396; doi:10.1371/journal.pone.0213349)
Supplement: S3 Table — (PDF) [file pone.0213349.s003.pdf]

S3 Table. Results from MLM with techno-stressors and social coping as mediators.

Within Level

Outcome: social coping

	Estimate	SE	*p*	LL	UL	R^2	*p*
tstr	.116	.032	.000	.064	.168	.021	.016
dep	.061	.028	.029	.015	.107		

Outcome: technology-related strain

	Estimate	SE	*p*	LL	UL	R^2	*p*
tstr	.290	.034	.000	.235	.290	.093	.000
social	.081	.027	.002	.037	.124		
dep	-.024	.035	.488	-.081	.033		

Between Level

Outcome: techno-stressors

	Estimate	SE	*p*	LL	UL	R^2	*p*
age	-.038	.031	.221	-.088	.013	.125	.000
dep	.348	.029	.000	.300	.397		

Outcome: social coping

	Estimate	SE	*p*	LL	UL	R^2	*p*
age	-.087	.030	.003	-.137	-.038	.284	.000
tstr	.168	.035	.000	.110	.225		
dep	.433	.035	.000	.376	.490		

Outcome: technology-related strain

	Estimate	SE	*p*	LL	UL	R^2	*p*
age	-.065	.024	.006	-.104	-.026	.618	.000
tstr	.785	.021	.000	.750	.820		
social	.030	.033	.372	-.025	.084		
dep	-.049	.029	.097	-.097	.000		

Total, Direct and Indirect Effects

age to strain

	Estimate	SE	*p*	LL	UL
total	-.122	.033	.000	-.176	-.068
total indirect	-.057	.025	.022	-.097	.016
via tstr	-.029	.024	.222	-.069	.010
via social	-.003	.003	.394	-.008	.002
via tstr and social	.000	.000	.447	-.001	.000
direct	-.065	.024	.006	-.104	-.026

Note. Model was fit using Mplus 7.1. *N* = 1,216, SE = standard error, *p* = two-tailed *p*-value, LL/ UL = 95% lower-level and upper-level confidence interval, social = social instrumental support, tstr = techno-stressors
