# Supplementary material for: The mediating role of coping behavior on the age-technostress relationship: A longitudinal multilevel mediation model
Source: PLoS One. 2019 Mar 5;14(3):e0213349. doi: 10.1371/journal.pone.0213349 (PMC6400396; doi:10.1371/journal.pone.0213349)
Supplement: S4 Table — (PDF) [file pone.0213349.s004.pdf]

S4 Table. Results from MLM with techno-stressors and behavioral disengagement as mediators.

**Within Level**							
**Outcome: behavioral disengagement**							
	Estimate	SE	*p*	LL	UL	R^2	*p*
tstr	.225	.035	.000	.167	.282	.047	.001
dep	-.053	.030	.072	-.102	-.005		

**Outcome: technology-related strain**							
	Estimate	SE	*p*	LL	UL	R^2	*p*
tstr	.266	.032	.000	.213	.319	.107	.000
diseng	.147	.032	.000	.094	.200		
dep	-.012	.034	.717	-.067	.043		

**Between Level**							
**Outcome: techno-stressors**							
	Estimate	SE	*p*	LL	UL	R^2	*p*
age	-.037	.031	.226	-.087	.013	.126	.000
dep	.349	.029	.000	.301	.398		

**Outcome: behavioral disengagement**							
	Estimate	SE	*p*	LL	UL	R^2	*p*
age	-.114	.026	.000	-.067	.007	.569	.000
tstr	.782	.025	.000	.741	.823		
dep	-.168	.030	.000	-.217	-.119		

**Outcome: technology-related strain**							
	Estimate	SE	*p*	LL	UL	R^2	*p*
age	-.030	.022	.175	-.067	.007	.665	.000
tstr	.531	.050	.000	.449	.613		
diseng	.331	.055	.000	.241	.421		
dep	.021	.024	.380	-.018	.021		

**Total, Direct and Indirect Effects**					
**age to strain**					
	Estimate	SE	*p*	LL	UL
total	-.122	.033	.000	-.176	-.068
total indirect	-.091	.027	.001	-.136	-.046
via tstr	-.020	.016	.226	-.046	.007
via diseng	-.038	.011	.000	-.055	-.020
via tstr and diseng	-.010	.008	.245	-.023	.004
direct	-.030	.022	.175	-.067	.007

Note. Model was fit using Mplus 7.1. *N* = 1,216, SE = standard error, *p* = two-tailed *p*-value, LL/ UL = 95% lower-level and upper-level confidence interval, diseng = behavioral disengagement, tstr = techno-stressors
