# Supplementary material for: The mediating role of coping behavior on the age-technostress relationship: A longitudinal multilevel mediation model
Source: PLoS One. 2019 Mar 5;14(3):e0213349. doi: 10.1371/journal.pone.0213349 (PMC6400396; doi:10.1371/journal.pone.0213349)
Supplement: S5 Table — (PDF) [file pone.0213349.s005.pdf]

S5 Table. Spiral Effects: results from two serial mediation models.

Model 1 (age → t_1 strain → t_2 diseng → t_3 strain)			
	**t_1 strain**	**t_2 diseng**	**t_3 strain**
	R^2 = .05***	R^2 = .25***	R^2 = .54***
	β [95 % CI]	β [95 % CI]	β [95 % CI]
age	-.01 [-.02; .00]	-.01 [-.01; .00]	-.01 [-.02; .00]
t_1 dep (control)	.26 [.16; .35]	-.01 [-.06; .03]	.10 [-.02; .00]
t_1 strain (M1)		.22 [.19; .25]	.65 [.03; .17]
t_2 diseng (M2)			.34 [.19; .48]
**direct effect**			
age → strain t_3			-.01 [-.01; -.00]
**indirect effects**			
age → t_1 strain → t_3 strain			-.01 [-.01; .00]
age → t_1 strain → t_2 diseng → t_3 strain			-.00 [-.00; .00]
age → t_2 diseng → t_3 strain			-.00 [-.01; .00]

Model 2 (age → t_1 diseng → t_2 strain → t_3 diseng)			
	**t_1 diseng**	**t_2 strain**	**t_3 diseng**
	R^2 = .02***	R^2 = .22***	R^2 = .37***
	β [95 % CI]	β [95 % CI]	β [95 % CI]
age	-.01 [-.01; -.00]	-.01 [-.02;-.00]	-.00 [-.01; .00]
t_1 dep (control)	.06 [.01; .11]	.23 [.14; .32]	-.01 [-.05; .03]
t_1 diseng (M1)		.82 [.67; .96]	.43 [.36; .50]
t_2 strain (M2)			.13 [.10; .17]
**direct effect**			
age → diseng t_3			-.00 [-.01; -.00]
**indirect effects**			
age → t_1 disng → t_3 diseng			-.00 [-.01; .-00]
age → t_1 diseng → t_2 strain → t_3 diseng			-.00 [-.00; -.00]
age → t_2 strain → t_3 diseng			-.00 [-.00; -.00]

Note. *SE* = standard error, * = *p*-value < .05, ** = *p*-value < .01, *** = *p*-value < .001, dep = IT-dependency, diseng = behavioral disengagement, strain = technology-related strain, CI = 95% confidence intervals based on bias corrected bootstrap analyses with 1,000 repetitions
